# Efficacy of Oral Probiotic Supplementation in Preventing Vulvovaginal Infections During Pregnancy: A Randomized and Placebo-Controlled Clinical Trial

**DOI:** 10.3390/nu16244406

**Published:** 2024-12-22

**Authors:** Enav Yefet, Abeer Suleiman, Raul Colodner, Shlomo Battino, Malak Wattad, Olga Kuzmin, Zohar Nachum

**Affiliations:** 1 Department of Obstetrics and Gynecology, Tzafon Medical Center, Poriya 1528001, Israel; 2 Women’s Health Center, Clalit Health Services, Afula 1834111, Israel; 3 Azrieli Faculty of Medicine, Bar-Ilan University, Safed 1311502, Israel; 4 The Holy Family Medical Center, Nazareth 1641100, Israel; 5 Department of Obstetrics and Gynecology, Emek Medical Center, Afula 1834111, Israel; 6 Microbiology Laboratory, Emek Medical Center, Afula 1834111, Israel; 7 Ruth and Bruce Rappaport Faculty of Medicine, Technion—Israel Institute of Technology, Haifa 3109600, Israel

**Keywords:** pregnancy, probiotics, vulvovaginal infections, bacterial vaginosis, abnormal vaginal flora, vulvovaginal candidiasis, prevention

## Abstract

Background/Objective: This study aimed to investigate the efficacy of oral probiotic supplementation in preventing vulvovaginal infections (VVIs) in pregnant women, specifically focusing on abnormal vaginal flora (AVF), bacterial vaginosis (BV), and vulvovaginal candidiasis (VVC). Methods: A multicenter-prospective-randomized, double-blind, placebo-controlled trial was conducted during 2016–2019. Women with normal vaginal flora (Nugent score < 4 and no candida) were divided into a research group, receiving 2 capsules/day of oral probiotic formula containing *Bifidobacterium bifidum*, *Bifidobacterium lactis*, *Lactobacillus acidophilus*, *Lacticaseibacillus paracasei*, *Lacticaseibacillus rhamnosus*, and *Streptococcus thermophilus*, or a control group, receiving a placebo until delivery. Once a month and following complaints, a vaginal smear was taken to assess vaginal flora. Vaginal colonization with the specific lactobacilli from the probiotic capsules was detected using the matrix-assisted laser desorption/ionization time-of-flight mass spectrometry. The primary outcome was the rate of women who developed VVI. Results: Forty-nine and fifty-one women were analyzed in the probiotic and placebo cohorts, respectively. There was no difference in the rate of VVI between probiotic and placebo groups (14 (29%) versus 14 (27%), respectively; *p* = 0.80). No woman had vaginal colonization with lactobacilli from the probiotic capsule. Conclusions: The tested oral probiotic product did not reduce the rate of VVI in pregnant women with normal vaginal flora.

## 1. Introduction

Vulvovaginal infections (VVIs) are a common medical problem. The most prevalent VVI is bacterial vaginosis (BV) or its milder form, abnormal vaginal flora (AVF), which can be found in 30% of pregnant women [1]. In these conditions, the normal lactobacilli-containing flora are replaced by anaerobic bacteria [2]. The second most common infection is caused by *Candida* species, with *Candida albicans* being the most frequent yeast infection causing vulvovaginal candidiasis (VVC) [3].

During pregnancy, VVIs have been shown to cause serious maternal and neonatal complications. BV has been associated with an increased risk of postabortal pelvic inflammatory disease, abnormal cervical cytology, premature rupture of membranes (PROM), preterm labor and delivery, chorioamnionitis, and post-cesarean endometritis [4]. VVC is more prevalent during pregnancy, affecting up to 10% of women [5]. Recurrences are more common, and therapeutic response is reduced compared to non-pregnant women, probably due to hormonal and immunologic alterations during pregnancy [3]. Maternal VVC during the third trimester has been associated with vertical transmission of yeast infection, causing oral thrush and diaper dermatitis in infants during the first year of life [6].

Antibiotic treatment for BV/AVF is usually the treatment of choice for this infection and has been shown to decrease the rate of late abortions and preterm deliveries [7]. However, in 25% of cases, antibiotic treatment fails to eradicate AVF/BV, and the recurrence rate is substantial [1].

Lactobacilli are the main component of the vaginal flora and are known to protect the vagina against pathogens. The mechanisms by which lactobacilli protect the vagina against pathogenic infections are by producing antimicrobial compounds (e.g., hydrogen peroxide, lactic acid, bacteriocin-like substances) and the capability to adhere and compete for adhesion sites in the vagina [2]. Thus, it was suggested to use them as preventive and therapeutic measures in cases of VVIs. Several studies have investigated this hypothesis with encouraging results in pregnant and non-pregnant women [8]. In a systematic review, which analyzed 24 clinical trials and 8242 participants, probiotics were shown to reduce the levels of IL-6 and IL-1β, as well as the overall Amsel’s criteria and Nugent score for restitution of a balanced vaginal microbiota. In addition, in subjects treated with probiotics, BV cure rates were higher than those in women treated with antibiotics without additional adverse events [8].

Studies on the effect of oral probiotic supplements on VVIs during pregnancy have demonstrated conflicting results with considerable methodological variations and limitations [8]. These limitations included the use of heterogeneous study populations with normal vaginal flora and VVI, lack of evaluation of vaginal colonization by specific probiotic strains, different methods for VVI diagnosis, short treatment duration, and lack of evaluation of VVC prevention [8]. The effect of probiotics following antibiotic or antimycotic treatment in cases where VVI occurred during the follow-up period was not studied.

This randomized controlled trial evaluated the efficacy of oral probiotic supplementation in preventing AVF, BV, and VVC among pregnant women with initially normal vaginal microbiota, constituting a primary prevention strategy.

## 2. Material and Methods

A multicenter, double-blind, randomized, placebo-controlled trial was conducted between November 2016 and December 2019 in Israel at three university-affiliated sites: Emek Medical Center in Afula, the Holy Family Medical Center in Nazareth, and the Women’s Health Center of Clalit Health Services in Afula. Participants enrolled from the medical centers were ambulatory and recruited through the medical centers’ outpatient clinics. The study was authorized by the review boards of the participating centers (ClinicalTrials.gov identifier: NCT02795845). Participants provided written informed consent.

Pregnant women above 18 years old up to their 30th week of gestation, who were tested for VVI due to vulvovaginal symptoms (vaginal discharge, pruritus, burning sensation, dryness, and erythema), were assessed for eligibility. The presence of AVF/BV/VVC was assessed by taking a vaginal smear. Women were considered eligible if both AVF/BV (Nugent score < 4) and candida were negative in direct microscopy [9]. We chose to test for eligibility women who complained of vaginal symptoms for the following reasons: (1) in those women, vaginal smear is indicated to evaluate VVI and treatment accordingly, (2) symptomatic women are the target population for probiotic treatment if proved effective and not asymptomatic women, and (3) since vaginal examination and obtaining vaginal smear are accompanied by considerable discomfort together with the fact that vaginal complaints are unlikely to affect the reliability of the vaginal smear results, it seemed more ethical to use this population of women for this study.

Exclusion criteria included preterm PROM at enrollment, immunocompromised women (e.g., active autoimmune diseases, chronic immunosuppressive drug use), trichomonas infection at enrollment, and allergy to soy or fish (since the study product capsules were manufactured in the same line as soy and fish). Women who took probiotic treatments and refused to discontinue treatment were also excluded.

### 2.1. Randomization and Masking

Participating women were randomly assigned (1:1) to treatment groups using a computer randomization sequence generation program with a block size of four. The randomization code was stored in sealed opaque envelopes until intervention was assigned by the study physicians.

### 2.2. Interventions

Women were allocated to receive one capsule twice-a-day until delivery of either oral probiotic formula containing *Bifidobacterium bifidum* Bb-06, *Bifidobacterium lactis* Bi-07, *Lactobacillus* (L.) *acidophilus* La-14, *Lacticaseibacillus paracasei* Lpc-37, *Lacticaseibacillus rhamnosus* Lr-32, and *Streptococcus thermophilus* St-21 (>6 × 10^9^ CFU/capsule; Manufacturer: Danisco, USA Inc., Thomson, IL, USA, batch number 61002442) or placebo that looked identical to the probiotic capsules in the same containers. All those bacteria were shown to be safe during pregnancy [8,10] and to either colonize the normal vaginal flora [8,11,12] and/or to be a part of a probiotic formula that improved vaginal dysbiosis [13,14,15,16].

Before the trial initiation, we cultured the lactobacilli from a sample of the probiotic capsules to ensure they were viable and could be detected by our culture techniques.

Once a month and in case of symptoms consistent with VVI, the following were evaluated: clinical evaluation of signs and symptoms for VVI; vaginal smear for AVF/BV/VVC; possible adverse effects and compliance that was assessed by counting the remaining capsules that were returned by the patients. The participants were instructed not to consume other probiotic supplements.

In cases of VVI, the women were treated with antibiotics for BV/AVF (either oral metronidazole 500 mg twice-daily or clindamycin 300 mg twice-daily for seven days) or vaginal tab of clotrimazole 500 mg twice-a-week for one week for VVC. During that time, the study products were continued until delivery.

### 2.3. Semi-Quantitative Assessment of Vaginal Lactobacilli Colonization

Before the initial treatment with either probiotic capsule or placebo and after 1–2 months and 3–4 months of treatment, a vaginal sample was obtained for bacterial culture, in which a semi-quantitative assessment of the amount of vaginal lactobacilli was conducted, using selective culture plate for this strain and the specific lactobacilli from the probiotic capsules product was searched at each group [17]. The pattern of bacterial growth was used for a semi-quantitative interpretation on a scale of 0 (no vaginal colonization) to 4 (substantial colonization).

### 2.4. Identifying the Specific Lactobacilli of the Probiotic Product

The cultures described in the previous section were used to identify the specific lactobacilli from the probiotic capsules using the matrix-assisted laser desorption/ionization time-of-flight mass spectrometry (MALDI-TOF-MS) as described previously [18,19].

### 2.5. Study Endpoints

The primary endpoint was the rate of women who developed any VVI during the study period until delivery. Women were included if they participated until at least the first visit (around one month). Secondary endpoints included the rate of women who developed either AVF/BV, VVC, or urinary tract infection (UTI) during the study period, the duration of time from the beginning of the study until the first episode of VVI occurred, and the rate of VVI at each study visit, which took place once every month until delivery. Data regarding treatment results for VVIs, obstetrical, and neonatal outcomes were also collected. Maternal adverse effects were documented, and the type of vaginal lactobacilli colonization was also assessed.

### 2.6. Statistical Analysis

BV and VVC during pregnancy are approximately 30% and 10%, respectively [1,5]. We estimated that this would be the appearance rate in the placebo group, and in the probiotic group the appearance rate would be 10% and 5%, respectively. Assuming a reduction from 40% (30% AVF/BV + 10% VVC) to 15% (10% AVF/BV + 5% VVC) in the appearance of VVI in the study group compared to control, a total of 98 women were required (80% power, 5% two-sided alpha).

Inter-cohort baseline characteristics and outcomes were compared using the Student’s *t*-test (or the Wilcoxon two sample test) for continuous variables and χ^2^ (or Fisher’s exact test) for categorical variables.

We evaluated the time from enrollment until the first VVI episode in each cohort by using the Kaplan–Meier curve in weeks. A log-rank test was performed to compare the groups’ survival curves.

Statistical analyses were carried out with SAS version 9.4 (SAS Institute, Cary, NC, USA). Significance was set at a *p* value < 0.05.

## 3. Results

**Figure 1:**
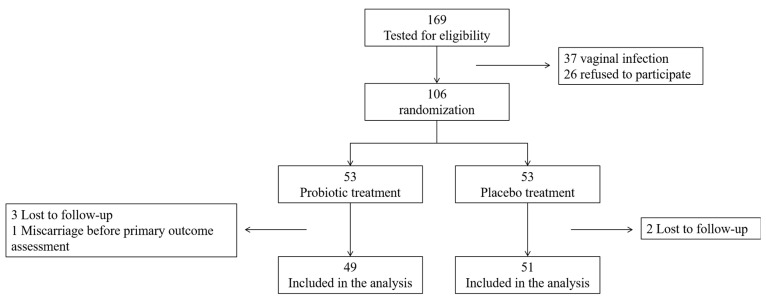
Patients’ flow chart.

A patients’ flow chart is presented in Figure 1. Overall, 49 and 51 women were allocated to the probiotic and placebo groups, respectively, and were included in the analysis.

Patients’ characteristics were comparable between the groups (Table 1).

Compliance with the study products consumption was high and comparable in both groups (mean consumption of 84 ± 29% versus 90 ± 18% of the capsules in the probiotic and the placebo group, respectively; *p* = 0.87).

**Figure 2:**
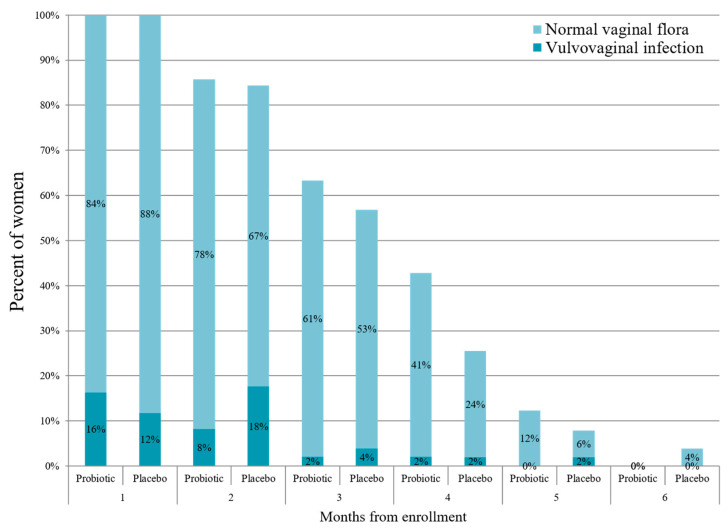
The rate of vulvovaginal infections (VVI) throughout the study in the probiotic (N = 49) and placebo (N = 51) groups. The study participants were invited for vaginal swab once in every month until delivery. Repeated vaginal swabs for verifying VVI eradication are not presented. *p* > 0.05 for all the time points.

**Figure 3:**
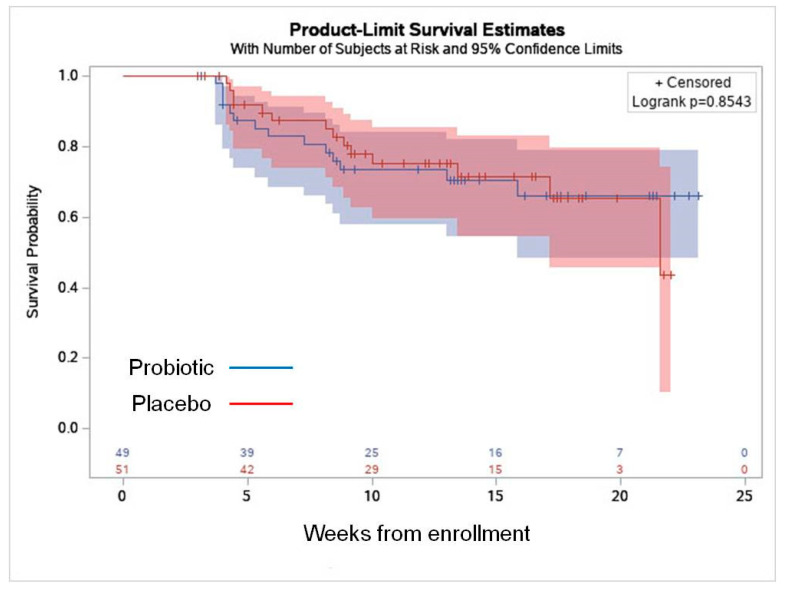
Kaplan–Meier survival curve representing the time from enrollment to the first vulvovaginal infection.

Mean study duration was 15.7 ± 5.2 and 15.1 ± 5.3 weeks in the probiotic and placebo groups, respectively (*p* = 0.59). There was no statistically significant difference between the groups in the rate of VVI, AVF/BV, VVC, or the rate of UTIs (Table 2). Time until first infection appearance was comparable between the groups (Table 2 and Figure 2). The rate of VVI at each visit is presented in Figure 3. There was no statistical difference between the probiotic and placebo groups in all the time points (*p* > 0.05 for all the comparisons).

**Figure 4:**
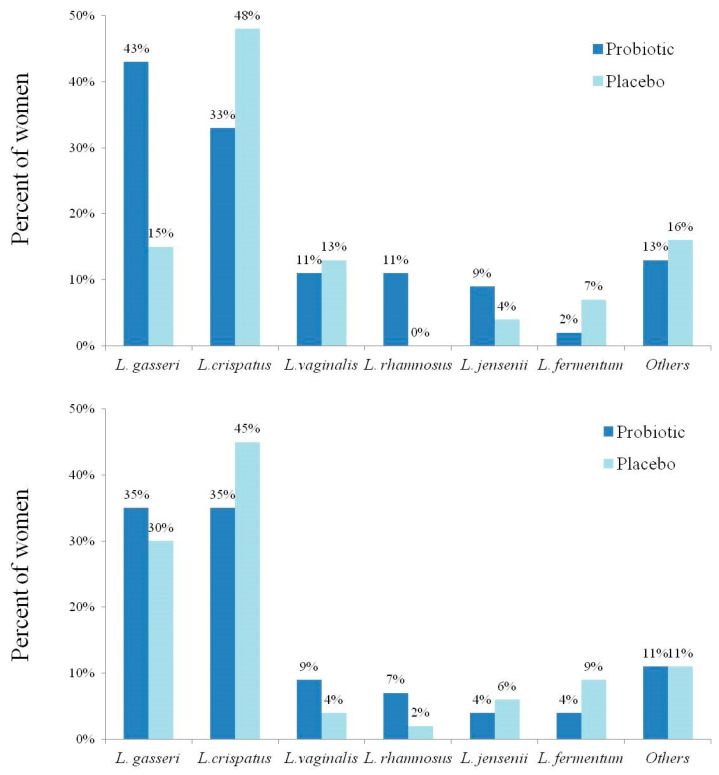
Percentage of vaginal lactobacilli (L.) strains from the study participants according to the study groups (46 and 45 women in the probiotic and placebo groups, respectively). Data from baseline (**lower panel**) and the last vaginal culture (**upper panel**) are presented. Regarding culture from the last visit, in 11 and 5 women, 2 L. strains were identified in culture in the probiotic and placebo groups, respectively. Two women in each group had 3 L. strains. The strains are listed in all the appropriate places. Comparison of the bacterial composition between both groups was not statistically significant (*p* = 0.06).

Pregnancy outcomes were comparable between the groups (Table 2). Semi-quantitative assessment of vaginal lactobacilli colonization demonstrated substantial growth in both groups (Table 2). The dominant lactobacilli identified using MALDI-TOF-MS from the last available vaginal specimens in each group are presented in Figure 4. No woman was found to have vaginal colonization of the specific bacterial strains of the probiotic supplements in all the available specimens that were cultured.

Subjective symptoms and objective vaginal examination at baseline, one month following treatment with the study product, and at the last visit of the study, are presented in Table 3. There was no difference between the groups at any of the time points except from pruritus, which was reported less in the probiotic group at the last visit (*p* = 0.01, Table 3).

Data regarding the treatment results of the VVI events are presented in Table 4. In the probiotic group, all the VVI events (N = 13) were resolved after one or two treatment cycles. In the placebo group, 11/12 (92%) VVI events were resolved.

Positive finding was considered at least mild severity.

Four (8%) and two (4%) women reported adverse effects in the probiotic and placebo groups, respectively (*p* = 0.43). In the probiotic group, the women reported gastrointestinal symptoms (abdominal pain, nausea, bloating, and diarrhea). In the placebo group, one woman reported continuous headache and the other one heartburn. Neonatal outcomes were comparable between the groups (Table 5).

## 4. Discussion

### 4.1. Principal Findings

In the present study, we investigated the effect of oral probiotic administration in preventing AVF/BV/VVC in pregnant women with normal vaginal flora. We found that the rate of VVI in this study was approximately 30%, consistent with the literature [1], and was not reduced in the probiotic treatment group compared to placebo. The time to infection and pregnancy and neonatal outcomes were not significantly different between the groups. Notably, the specific lactobacilli strains from the probiotic capsules were not detected in the vagina, despite the probiotics being taken for an average of 4 months.

### 4.2. Results in the Context of What Is Known

For this study, we chose to administer probiotics orally. Previous research has shown that oral intake of Lactobacillus strains can improve the vaginal microbial pattern in non-pregnant populations [8,16,20]. Studies on the effect of probiotics on VVI during pregnancy have yielded inconsistent results; *L. rhamnosus* and *L. reuteri* given orally did not substantially colonize the vagina or affect the prevalence of VVIs [21,22,23] or preterm births [24]. Conversely, oral administration of *L. rhamnosus* and *L. reuteri* decreased the rate of group B streptococcus (GBS) in pregnant women who were GBS carriers. However, transfer of the specific strains from the probiotic capsules was not assessed [25].

The similar rate of VVI in both groups of the current study was accompanied by the absence of the specific lactobacilli strains from the probiotic capsule in the vagina. This finding contrasts with previous studies conducted on non-pregnant women, in which *L. acidophilus*, *L. rhamnosus*, and *L. paracasei* were shown to colonize the vagina following oral administration, even after shorter treatment periods [8,11,12]. Although the species in these studies are the same as in ours, the strains differ. Our results are consistent with previous studies that demonstrated ineffective transfer of probiotics via the oral-vaginal route in pregnancy [21,22,26]. The inability to detect these bacteria is unlikely related to the MALDI-TOF-MS technique, as it has been shown to be highly effective for detecting specific bacterial strains in various specimens, including lactobacilli from the vagina [18,27,28]. Possible explanations for the difference in lactobacilli colonization between pregnant and non-pregnant women include ineffective transfer of probiotics from the gastrointestinal tract to the vagina due to decreased motility during pregnancy, and possible indirect mechanisms such as immunomodulation in the gastrointestinal tract. Another hypothesis is that in studies enrolling women with BV/AVF, the rate of lactobacilli colonization was higher than in women with normal vaginal flora [8,11,12,26] due to the presence of BV/AVF, where lactobacilli might better colonize the vagina than when it is colonized with other native lactobacilli. A healthy and stable vaginal microbiota has previously been observed to withstand probiotic colonization [29]. However, studies examining the effect of probiotics on pregnant women with BV/AVF did not demonstrate favorable effects [23,24].

### 4.3. Clinical and Research Implications

Fewer women reported pruritus in the probiotic group at the last study visit. Since this was the only difference between the groups, further investigation is needed to assess whether it is a true effect or potentially related to chance.

To the best of our knowledge, this is the first study to investigate the effect of probiotics for primary prevention of VVIs in pregnancy. Other studies examined the effect of probiotics on heterogeneous pregnant populations [21,22] or women with BV/AVF [23,24]. Another difference in our study is the use of antibiotics and antimycotic treatments to treat VVIs. This was conducted because the women were symptomatic and VVI developed under treatment with the study products. Importantly, while most studies used *L. rhamnosus* and *L. reuteri* and did not demonstrate favorable effects, we used *L. rhamnosus* and additional strains associated with positive effects on the vaginal microbiome and pregnancy outcomes. *Bifidobacterium* was highly represented in pregnant women with a normal cervical length [13]. *L. paracasei* demonstrated an inhibitory effect on pathogenic Gram-positive and Gram-negative bacteria, *Candida albicans*, and *Candida krusei* [30]. Probiotic formulas containing *L. rhamnosus* and *L. paracasei* decreased vulvovaginal symptoms and improved vaginal dysbiosis [8,14]. Future studies should elucidate whether the current probiotic formula will be effective for VVI prevention in non-pregnant women and whether probiotic formulas containing other strains will be effective for VVI prevention during pregnancy.

### 4.4. Strengths and Limitations

The strengths of this study include its multicenter randomized placebo-controlled design, high compliance rate, use of methods to detect specific bacterial strains from the capsule, and investigation of primary prevention of VVI in pregnancy as a primary outcome in a homogeneous population, which had not been studied previously.

A limitation of the study is a certain degree of heterogeneity in the study population, as three VVIs were included, AVF, BV, and VVC, with BV treated using one of two antibiotics. The study was not powered to evaluate pregnancy and neonatal outcomes, and the results are specific to the probiotic product used. This product was chosen because it contained bacterial strains with beneficial effects on the vaginal microbiome and was commercially available, allowing for easy purchase if positive effects were demonstrated.

## 5. Conclusions

The results of this randomized controlled trial indicate that the oral probiotic formulation evaluated in our study did not demonstrate a statistically significant reduction in the incidence of VVIs among pregnant women who initially presented with normal vaginal flora. This finding suggests that the primary prevention strategy of administering oral probiotics to maintain vaginal health during pregnancy may not be as effective as hypothesized.

However, a noteworthy observation emerged from our analysis: women in the probiotic group reported a lower incidence of pruritus compared to the control group. While this outcome was not the primary focus of our investigation, it presents an intriguing avenue for future research. The potential relationship between probiotic supplementation and reduced vaginal discomfort warrants further exploration, as it could have implications for improving the quality of life for pregnant women.

## Figures and Tables

**Table 1:** Baseline patients’ characteristics.

Characteristics	Probiotic N = 49	Placebo N = 51	*p* Value
Maternal age, years	31.4 (5.7) [31, 28–35]	31.3 (4.9) [32, 28–34]	0.87
Pre-pregnancy BMI, kg/M^2^	23.3 (4.2) [22.8, 20.6–24.7]	24.3 (4.1) [23.7, 21.9–26.9]	0.25
Current BMI, kg/M^2^	25.6 (4.1) [25.4, 22.6–27.8]	26.7 (4.4) [25.9, 23.6–30.1]	0.18
Current weight, kg	67.2 (10.6) [65, 61–71]	71.6 (13.4) [70, 62–80]	0.07
Pregnancy number	2.9 (1.6) [3, 2–4]	3.1 (1.8) [3, 2–4]	0.82
Num. of previous births	1.3 (1.2) [1, 0–2]	1.3 (1.2) [1, 0–2]	0.84
Risk factor for preterm birth	26 (53%)	19 (37%)	0.11
Num. of previous preterm births	0.2 (0.5) [0, 0–0]	0.1 (0.4) [0, 0–0]	0.51
Gestational week at beginning of study treatments	22.2 (5.2) [20.7, 18.9–25.9]	23.1 (5.2) [23, 19.1–28.1]	0.38
Cigarette smoking before pregnancy	10 (20%)	9 (18%)	0.73
Current cigarette smoking	4 (8%)	5 (10%)	1
Marital status: Married	49 (100%)	48 (94%)	0.5
Years of Education	15.2 (2.5) [16, 12–17]	14.3 (2.7) [15, 12–16]	0.04
Probiotic food in the diet	1 (2%)	3 (6%)	0.62
Regular antibiotic use prior to inclusion	2 (4%)	0 (0%)	0.24
Antimycotic use during pregnancy prior to inclusion	2 (4%)	2 (4%)	1
UTI during pregnancy prior to inclusion	9 (18%)	5 (10%)	0.22
Num. of UTI events during pregnancy prior to inclusion	0.2 (0.6) [0, 0–0]	0.1 (0.4) [0, 0–0]	0.21
Vaginal pH > 5	4 (8%)	4 (8%)	1
Multiple gestation	9 (18%)	11 (22%)	0.69
Gestational diabetes mellitus	6 (12%)	6 (12%)	0.94
Pre-eclampsia/gestational hypertension	5 (10%)	2 (4%)	0.26

Values are presented as mean (standard deviation) [median, IQR], or number (percent). Abbreviations: BMI, body mass index; UTI, urinary tract infection.

**Table 2:** Study endpoints—maternal outcomes.

Outcomes	Probiotic N = 49	Placebo N = 51	*p* Value
VVI *	14 (29%)	14 (27%)	0.80
VVC	3 (6%)	4 (8%)	1
BV/AVF	14 (29%)	13 (25%)	0.64
Time until first infection (weeks)	6.9 (3.7) [5.6, 4.0–8.4]	9.0 (5.2) [8.3, 4.4–10.0]	0.13
Gestational week at first infection **	27.4 (4.0) [26.5, 24.0–30.0]	30.9 (5.8) [32.5, 26.0–36.0]	0.10
Recurrent event of VVI ***	1 (2%)	4 (8%)	0.36
UTI event	3 (6%)	4 (8%)	1
Substantial vaginal lactobacilli colonization ^£^	40 (89%)	35 (78%)	0.16
Delivery week	37.9 (2.5) [38.4, 37.3–39.0]	38.2 (2.0) [38.4, 37.0–39.6]	0.65
Preterm delivery	7 (14%)	10 (20%)	0.48
Delivery mode: Vaginal	35 (71%)	32 (63%)	0.67
Vacuum	2 (4%)	2 (4%)	
Cesarean delivery	12 (24%)	17 (33%)	
PPROM	3 (6%)	5 (10%)	0.72
Chorioamnionitis	1 (2%)	0 (0%)	0.49
Intrapartum fever	1 (2%)	1 (2%)	1
Endometritis	0 (0%)	1 (2%)	1
Study duration (weeks)	15.7 (5.2) [16.1, 11.6–19.6]	15.1 (5.3) [14.7, 11.0–19.3]	0.59

Values are presented as mean (standard deviation), [median, IQR], or number (percent). One woman had late abortion. Neonatal outcomes are missing in this case. * At least one episode. ** Refers to women who had vaginal infection during the study. *** In the placebo group, one woman had two recurrences. ^£^ Refers to score 3 and 4 at semi-quantitative assessment of vaginal lactobacilli colonization in the last culture available (N = 45 women in each group). Abbreviations: AVF, abnormal vaginal flora; BV, bacterial vaginosis; PPROM, preterm premature rupture of membrane; UTI, urinary tract infection; VVC, vulvovaginal candidiasis; VVI, vulvovaginal infection.

**Table 3:** Vulvovaginal symptoms and signs.

	Baseline			One Month			End of the Study		
Outcomes	Probiotic N = 49	Placebo N = 51	*p* Value	Probiotic N = 49	Placebo N = 51	*p* Value	Probiotic N = 49	Placebo N = 51	*p* Value
Subjective vulvovaginal symptoms	49 (100%)	51 (100%)		37 (76%)	44 (86%)	0.17	41 (84%)	42 (82%)	0.86
Vaginal discharge severity: None	0	0	0.58	13 (27%)	11 (22%)	0.54	8 (16%)	11 (22%)	0.56
Mild	15 (31%)	11 (22%)		12 (24%)	18 (35%)		19 (39%)	15 (29%)	
Moderate	27 (55%)	31 (61%)		20 (41%)	16 (31%)		17 (35%)	16 (31%)	
Severe	7 (14%)	9 (18%)		4 (8%)	6 (12%)		5 (10%)	9 (18%)	
Pruritus	18 (37%)	22 (43%)	0.51	12 (24%)	11 (22%)	0.73	4 (8%)	14 (27%)	0.01
Burning sensation	6 (12%)	8 (16%)	0.62	2 (4%)	3 (6%)	1	3 (6%)	3 (6%)	0.96
Dryness	3 (6%)	7 (14%)	0.32	3 (6%)	3 (6%)	1	2 (4%)	4 (8%)	0.68
Erythema	3 (6%)	6 (12%)	0.49	0 (0%)	3 (6%)	0.24	1 (2%)	2 (4%)	1
Objective Vaginal discharge	47 (96%)	46 (90%)	0.44	36 (73%)	31 (61%)	0.18	35 (71%)	38 (75%)	0.73
Objective Vulvovaginal erythema	1 (2%)	1 (2%)	1	2 (4%)	1 (2%)	0.61	1 (2%)	1 (2%)	1

**Table 4:** Response to treatment of VVI events.

	Eradication	Probiotic	Placebo
VVI	Spontaneous	1	0
	After one treatment cycle	8	9
	After two treatment cycles	4	2
	No eradication after two treatment cycles	0	1
	Unknown *	2	7
AVF/BV	Spontaneous	1	0
	After one treatment cycle	9	9
	After two treatment cycles	3	1
	No eradication after two treatment cycles	0	1
	Unknown *	2	6
VVC	Spontaneous	0	0
	After one treatment cycle	3	2
	After two treatment cycles	0	0
	No eradication after two treatment cycles	0	0
	Unknown *	0	3

Number of VVIs (AVF/BV and VVC) is presented. One and four women in the probiotic and placebo groups, respectively, had more than one VVI event and all of them are listed. Co-infection with both AVF/BV and VVC are listed in all the appropriate rows. In AVF/BV and VVC, eradication was considered the resolution of the infection that the treatment was targeted to. In VVI, eradication was considered a complete resolution to normal vaginal flora. * Delivered before treatment or before repeated vaginal smear was taken. Abbreviations: AVF, abnormal vaginal flora; BV, bacterial vaginosis; VVC, vulvovaginal candidiasis; VVI, vulvovaginal infection.

**Table 5:** Study endpoints—Neonatal outcomes.

Outcomes	Probiotic N = 58	Placebo N = 62	*p* Value
SGA	8 (14%)	5 (8%)	0.31
Apgar score at 1 min < 7	4 (7%)	2 (3%)	0.43
Apgar score at 5 min < 7	2 (3%)	1 (2%)	0.61
NICU admission	8 (14%)	5 (8%)	0.31
Neonatal sepsis	2 (3%)	0 (0%)	0.23
Neonatal RDS	3 (5%)	0 (0%)	0.11
IVH	2 (3%)	0 (0%)	0.23

Values are presented as number (percent). Abbreviations: IVH, intraventricular hemorrhage; NICU, neonatal intensive care unit; RDS, respiratory distress syndrome; SGA, small for gestational age.

## Data Availability

The data from this study is available from the corresponding author upon a reasonable request and following approval of the institutional review board.
